# Affording Social Experience for Adolescents Using Immersive Virtual Reality: A Moderated Mediation Analysis

**DOI:** 10.3390/children11111362

**Published:** 2024-11-09

**Authors:** Gege Li, Heng Luo, Xin Yin, Yan Zhang, Zijian Li

**Affiliations:** 1 Faculty of Artificial Intelligence in Education, Central China Normal University, Wuhan 430079, China; ligg323@mails.ccnu.edu.cn (G.L.); luoheng@mail.ccnu.edu.cn (H.L.); yinxinxin@mails.ccnu.edu.cn (X.Y.); zhangyan123@mails.ccnu.edu.cn (Y.Z.); 2 School of Fine Arts, Central China Normal University, Wuhan 430079, China

**Keywords:** IVR collaboration game, social experience, social–emotional competence, technology acceptance, presence, productive collaboration

## Abstract

Background: Immersive virtual reality (IVR) serves as a promising tool to provide adolescents with enriched social experience due to its high-fidelity simulations and multimodal interaction. This study aims to design and develop a multi-user IVR collaborative game utilizing simultaneous localization and mapping (SLAM)-based inside-out tracking technique to foster social experience among students. Also, this study explored the mechanism by which technology acceptance affected social experience in the IVR collaboration game, focusing on the mediating effects of presence, collective efficacy, and group effectiveness, as well as the moderating effect of social–emotional competence (SEC). Methods: A total of 104 seventh graders from a middle school in Central China participated in this study and completed the questionnaire. Finally, 87 valid questionnaire responses were retrieved. Results: The results revealed that technology acceptance both directly and indirectly influenced social experience. The mediation analysis revealed a key pathway influencing social experience: technology acceptance → presence → collective efficacy → group effectiveness → social experience. However, no moderating effect of SEC was found in the relationship between technology acceptance and social experience, group effectiveness, and social experience. Conclusions: Based on these results, more appropriate IVR interventions could be developed for social–emotional learning among children and adolescents.

## 1. Introduction

Social experience refers to the collective interactions, activities, and relationships that individuals engage in within a social context. This has been conceptualized as an individual’s desire for and experience in novel contexts [1]. Social experience provides individuals with an opportunity to observe others’ communication performances, as well as to engage in and refine interaction skills [1]. Adequate social experience can facilitate the development of empathy, communication competence, social identity, and cohesion. In contrast, a lack of social experience can result in alienation, low self-efficacy, low social–emotional competence (SEC), and mental health problems. Furthermore, the COVID-19 pandemic in 2020 severely reduced the quantity of social experience among children and adolescents due to social isolation, a lack of physical exercise, and insufficient relevant professional support [2,3]. Such issues persist in the post-pandemic era and underscore the pressing need for effective interventions to address them.

Immersive virtual reality (IVR) technology is a promising technological tool to solve these problems by delivering authentic and vivid social experiences, given its capacity to provide high-fidelity simulations, rich sensory stimuli, and multimodal interaction [4,5]. IVR shows potential for enriching individuals’ social experience due to its distinctive characteristics, including immersion, presence, empathy, and embodiment [6,7,8,9]. By providing individuals with a sense of authenticity, IVR can afford more natural interactions and emotional responses, thus enabling users to obtain improved social experience with enhanced engagement and embodied cognition [8,10]. IVR represents the enrichment and expansion of traditional environments, offering several potential advantages. First, IVR can provide students with a safe and controllable environment to rehearse social skills [11]. Second, compared to traditional settings, IVR shows the potential to provide students with individualized social-emotional experiences [12], especially for students who tend to be more introverted in real life. Moreover, the IVR environment enables more diverse social scenarios featured by multimodal interactions with peers, avatars, and the virtual world [13].

The IVR environment comprises three significant constructs: technology acceptance, presence, and productive collaboration, that are fundamental to its design. First, as an emerging technology, students’ technology acceptance directly influences their learning experience. Understanding the extent to which students accept IVR also contributes to the further design of IVR environments. Presence is another key construct for the individual’s experience in the IVR environment. This refers to how much the environment affords individuals a medium-induced sense of being in the environment, as characterized by vividness and interactivity. This is also called “tele-presence” in the literature [14]. Productive collaboration is another crucial construct that requires attention to collaborative task design in the IVR environment. The difficulty of collaborative tasks in fantasy scenarios can influence students’ self-efficacy. The interplay of these three constructs influences the generation of social experience.

There are two main limitations of IVR literature regarding social experience. First, there is a lack of design precedents for promoting social experience through multi-user collaboration, despite the significant potential for social interaction and communication [15]. Also, there may be some challenges in multi-user collaboration, such as differences in skills, technology experience, and learning ability. Technical difficulties such as a lack of physical cues and usability might also limit the simultaneous presence of multiple users [16]. Second, most research has focused on impact studies, investigating the effectiveness of IVR, and there has been a lack of research on the constructs of collaboration and their underlying mechanisms for promoting social experience.

To address this research gap, we designed a multi-user IVR game using the simultaneous localization and mapping (SLAM)-based inside-out tracking technique to create an environment for promoting the social experience among multiple users. A moderated mediation model was employed to elucidate the mechanism by which several social psychological factors influence social experience, thereby improving our understanding of the interrelationship between these factors in a multi-user IVR environment. The model considered presence, collective efficacy, and group effectiveness as single and serial mediators. SEC was also considered to examine its moderating effect on the relationship between technology acceptance and social experience, as well as between group effectiveness and social experience.

## 2. Literature Review and Research Hypothesis

### 2.1. Theoretical Frameworks

The present study was guided by the framework proposed by Kreijns et al. (2004) in sociable learning environments, which contains three key constructs: sociability, social presence, and pedagogical techniques [17]. Sociability refers to the social relationships between different rules and roles, which are able to facilitate the emergence of a social presence. Based on this, the collaborative game in our study comprises three roles: defender, attacker, and collector, which require positive interdependence to complete the task. Social presence refers to the degree of salience and consequence of interpersonal relationships. Pedagogical techniques can foster a sound social space, characterized by effective work relationships, collective efficacy, strong group cohesiveness, and belonging. Another theoretical framework that guided our research is the Dalgarno and Lee (2010) model of learning in virtual reality learning environments (VLE) [18]. The model contains three key constructs: construction of identity, sense of presence, and co-presence. Identity construction supports user representation in virtual reality (VR) environment, especially in avatars. The sense of presence and co-presence are psychological experiences created by representational fidelity. In this study, the terms social presence, sense of presence, and co-presence are collectively defined as “presence”. These affordances would lead to better user social experiences in experiential learning, contextual learning, and collaborative learning. Also, we focus on the impact of technology acceptance on social experience. The integration of these two frameworks would offer a more comprehensive insight into the mechanisms of the social experience in IVR.

### 2.2. Technology Acceptance and Social Experience

Acceptance of IVR is a critical factor influencing its effectiveness. In the educational context, students’ perception of the usefulness of IVR significantly affects their experiences [19]. These experiences not only encompass personal learning aspects, such as classroom engagement and attentiveness [20], but also extend to social experiences, including students’ interactions within the IVR setting [21].

Technology acceptance refers to students’ willingness to embrace new technology, which consists of perceived usefulness (PU), perceived ease of use (PEU), and behavioral intention to use (ITU) a technology [22,23]. PU is defined as the extent to which users believe that technology will enhance their performance. PEU is the degree to which users believe that using a technology is free of effort [22]. These two factors consequently predicate users’ ITU the technology [24]. Previous studies have demonstrated that physical discomfort or other technical issues may hinder a student’s ability to accept IVR, thus potentially resulting in decreased enjoyment or participation in social activities within the IVR environment [25].

**Figure 1:**
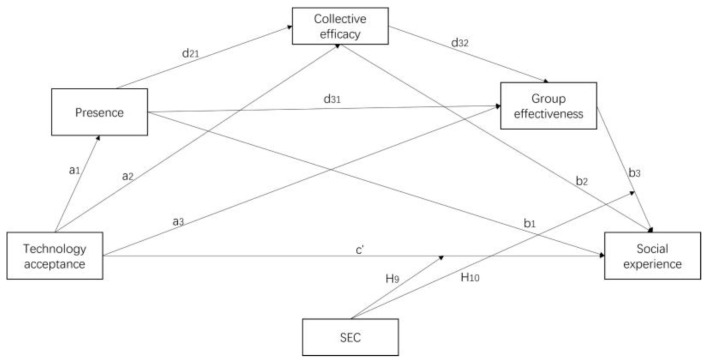
Moderated mediation model.

In technology-enhanced environments, the formation and quality of social experiences have been found to be correlated with users’ acceptance of technology. For instance, Lee et al. [26] introduced social network characteristics into the experience of a VR device and demonstrated that social interaction and the strength of social positively influenced users’ technology acceptance, which was mediated through perceived enjoyment. Magni and Pennarola [27] suggested that team-member interaction, organizational support, and affective commitment were significantly associated with PEU and PU. However, there have been a few research studies investigating social experience as the dependent variable. Given that multi-user IVR relies heavily on its immersive and interactive functions to enable group collaboration and communication, individuals who embrace the new technology tend to obtain better social experience [28,29]. We therefore proposed the following hypothesis (as seen in Figure 1):

**H1** **(c′):** Students’ technology acceptance positively affects their social experience.

### 2.3. Collective Efficacy and Group Effectiveness as Mediators

According to Wang and Hong [30], collective efficacy and group effectiveness are key elements of productive collaboration. They are also known to be closely related to social experiences [31,32,33]. Collective efficacy is defined as the shared belief of group members about the group’s ability to complete a specific task successfully or achieve a specific level of achievement; it is grounded in social cognitive theory [34,35]. Collective efficacy is closely linked to students’ social experience, as it can enhance social adaptability through augmented social support from family, friends, and teachers [36,37,38]. The collective efficacy of a group can be enhanced by PU, which can facilitate collaboration. Prior studies have indicated that the effective application of technology can contribute to a more positive attitude among students toward collaborative learning [39].

Another crucial aspect of productive collaboration examined in this study was group effectiveness. Group effectiveness refers to a group’s overall performance and efficiency in achieving goals and tasks. It encompasses the quality of task completion and communication between members [40]. Effective collaborative learning within groups can elicit more social support from members, thereby fostering students’ social adaptability and enhancing their social experience [41,42]. Group effectiveness can also be affected by technology acceptance. As an increase in PU and PEU among students can lead to higher engagement [43], improved learning efficiency [44], and enhanced knowledge sharing [45], it is evident that these factors can lead to more effective collaborative learning.

The interplay between collective efficacy and group effectiveness is essential for driving students’ learning experience in collaborative learning tasks. When team members believe in success, collective efficacy can motivate them to work harder, improve team collaboration and communication, and potentially improve team performance [46,47,48]. By fostering an environment where students believe in their collective capability, the overall effectiveness of group work can be enhanced, which leads to improved learning outcomes and a more enriched social experience. Therefore, we investigated the potential mediating roles of collective efficacy and group effectiveness in the relationship between technology acceptance and social experience, and proposed the following hypotheses (as seen in Figure 1):

**H2** **(a2b2):** Students’ collective efficacy mediates the relationship between technology acceptance and social experience.

**H3** **(a3b3):** Students’ group effectiveness mediates the relationship between technology acceptance and social experience.

**H4** **(a2d32b3):** Students’ collective efficacy and group effectiveness serially mediate the relationship between technology acceptance and social experience.

### 2.4. Presence, Antecedents, and Consequences

Presence is a crucial concept in virtual environments that shapes users’ overall immersive experience [49]. A high level of presence indicates a greater alignment between the user’s behavior in the virtual world and their actions in the real world, which facilitates knowledge transfer [50]. Rather than being regarded as a unitary concept, presence is differentiated into several sophisticated types, including physical and social presence [49,51,52]. Physical presence refers to the illusion of being in the virtual environment and occurs when individuals perceive and respond to the events in the virtual environment as though they were real [53,54]. Social presence is defined as the degree of interaction with agents and objects in the virtual environment and the resulting perception of interpersonal relationships [17,55]. In multi-user IVR, several people coexist in the same place simultaneously, which also increases the sense of co-presence as a type of social presence.

Presence is significantly enhanced by technology acceptance. Research studies have demonstrated that the sense of presence in IVR is greatly influenced by various technical factors, including technological immersion [56], multisensory feedback [57], and emotional and agency factors [58]. Altarteer and Charissis [59] also proved that a higher sense of presence is closely linked to the acceptance of IVR technology. Consequently, with superior PU and PEU, IVR could significantly enrich individuals’ perception of presence through enhanced immersion, interactivity, and imagination [60]. Individuals’ sense of presence also profoundly shapes the quality of productive collaboration. Feng et al. [61] investigated collaborative mechanisms in IVR and found that social presence was a crucial predictor of collective efficacy and social experience; this was consistent with the findings of Yoon and Leem [62] that social presence contributes to productive collaboration owing to its cohesive effect during interactions. Specifically, social presence positively enhances feelings of identity, belonging, and cohesion as a group, which are key elements fostering collective efficacy, group effectiveness, and social experience [40,63]. Hence, we therefore employed presence as a mediator and proposed the following hypotheses (as seen in Figure 1):

**H5** **(a1b1):** Students’ perceived presence mediates the relationship between technology acceptance and social experience.

**H6** **(a1d21b2):** Students’ perceived presence and collective efficacy serially mediate the relationship between technology acceptance and social experience.

**H7** **(a1d31b3):** Students’ perceived presence and group effectiveness serially mediate the relationship between technology acceptance and social experience.

**H8** **(a1d21d32b3):** Students’ perceived presence, collective efficacy, and group effectiveness are serial mediators in the relationship between technology acceptance and social experience.

### 2.5. Students’ Social–Emotional Competence (SEC) as a Moderator

SEC refers to individuals’ proficient handling of their intra- and interpersonal interactions. It has been recognized as an integral part of education and healthy adjustment in life for children and adolescents [64]. Two authoritative frameworks released by the Collaborative for Academic, Social, and Emotional Learning (CASEL) and the Organization for Economic Co-operation and Development (OECD) provide approaches for distilling and measuring SEC. CASEL [65] operationalizes SEC into five dimensions: self-awareness (awareness of one’s emotions, thoughts, and values); social awareness (understanding others’ perspectives and social norms); relationship skills (establishing and maintaining healthy and supportive relationships with others); responsible decision-making (making caring and constructive choices); and self-management (managing emotions, thoughts, and behaviors). Another framework has been used by the OECD and splits SEC into five personality factors [66]: conscientiousness, extraversion, emotional stability, openness, and agreeableness.

Researchers have emphasized the importance of involving mechanisms and manifestations of SEC and connecting them [64]. Previous research has indicated that SEC has an influential and moderating effect on technology acceptance [67,68]. A high level of SEC has been associated with a high level of perceived usefulness and behavioral intention. Compared with students with low levels of SEC, students with high SEC levels tend to show high technology acceptance, which can help them respond to challenges in social environments, thus gaining richer and more positive social experiences. SEC also requires a capacity to recognize others’ emotions and perspectives, as well as proficient communication and interaction skills [64]. Compared with students with low SEC levels, students with high SEC levels tend to show higher group effectiveness, which helps to enhance their social experience. Despite SEC, there are other factors like age, technology experience, and culture that may serve as moderating factors influencing social experience in IVR [69,70].

We therefore proposed the following hypotheses (as seen in Figure 1):

**H9:**  SEC moderates the relationship between technology acceptance and social experience.

**H10:**  SEC moderates the relationship between group effectiveness and social experience.

### 2.6. Moderated Mediation Model

Based on the two theoretical frameworks and our research hypothesis, we proposed the moderated mediation model (see Figure 1). First, as a technology alternative, it is necessary to understand students’ level of technological acceptance towards IVR. Moreover, IVR serves as a medium capable of constructing a social space for students, imbued with rich presence featured by social interaction and co-presence. Within this social space, students can collaboratively solve problems and share perspectives, leading to enhanced collective efficacy. This increase in collective efficacy, in turn, contributes to the improvement in group effectiveness. Ultimately, these factors collectively contribute to the enhancement of students’ social experience.

## 3. Design of the IVR Collaboration Game

**Figure 2:**
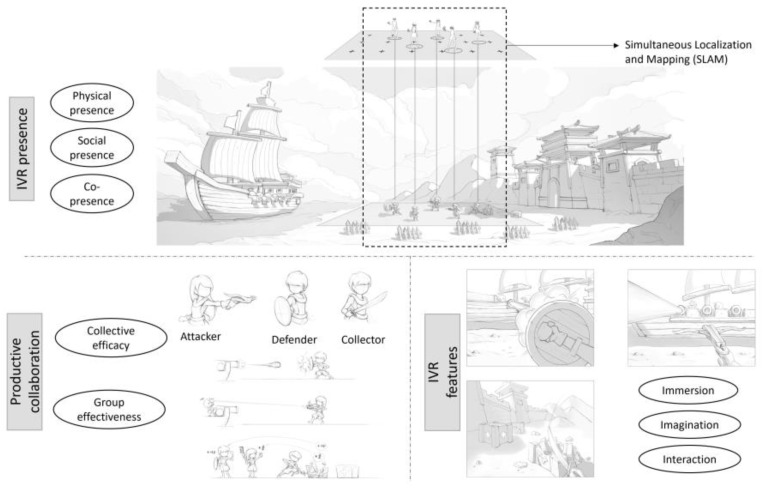
Design of the immersive virtual reality (IVR) collaboration game.

The IVR collaboration game used in this study was developed by the research team and is named The League of Castle Defenders. This collaboration game was designed and developed based on the theoretical frameworks of Kreijns et al. and Dalgarno and Lee [17,18], those frameworks indicate that a sociable technology-mediated environment should contain three key constructs: productive collaboration, presence, and technical features [49]. The main design decisions of the IVR game corresponding to the three key constructs are shown in Figure 2. The multi-user IVR game was inspired by the ancient Chinese legendary story, the heroine of Mulan, in which heroes need to cooperate to fight against the enemies’ artillery attack to protect their homeland. There are three roles in the multi-user IVR game: defender, attacker, and collector. The defender stands in front of the team to block the artillery attack. The attacker slays enemies with arrows while avoiding the artillery attack. The collector finds and opens supply boxes across a large area and provides defenders with blood or the attacker with arrows. They need to complete the mission collaboratively to achieve victory. We believe that the famous historical background engages the students with the three-hero role script promoting positive interdependence and group efficacy, and the athletic and strategic nature of the challenge offers opportunities for self-evaluation and collaborative problem-solving.

To achieve co-presence among multiple users in the IVR environment, inside-out tracking spatial positioning technology was used based on the SLAM algorithm. The students in the real world correspond to the characters in the virtual world. By leveraging SLAM algorithms and real-time synchronization to achieve multi-user IVR spatial positioning, combined with the mechanisms for rendering virtual scenes, the technology enables co-presence among multiple users within the virtual learning environment, thereby enhancing a sense of social presence.

Productive collaboration can be achieved with the immersive multi-user game in our study to create effective work relationships, strong group cohesiveness, and a strong sense of community. Players can freely select their roles based on their own interests and skills. The interdependence of the three roles can enhance individual efficacy, which in turn can facilitate further improvements in collective efficacy. We also revised the game mechanics and selected a level of difficulty that is most suitable for middle school students to complete based on a previous pilot study, which can further enhance the players’ group effectiveness.

The environmental design and development of the IVR game draw upon the three technical features of IVR: immersion, interaction, and imagination. The game provides a fully immersive virtual world in which students need to take some actions, including interacting with the virtual world and with their peers. These interactions can improve students’ sense of immersion and agency. The fantasy context and virtual characters in the game can also stimulate player engagement.

## 4. Method

### 4.1. Context and Participants

The study was conducted in Gong’an, a county in Central China. A total of 104 seventh graders from a middle school participated in this study. There were two main reasons for selecting the seventh-grade students at this middle school in the county to participate. First, Gong’an is a typical county in Hubei province; it is characterized by a mixed socioeconomic structure and above-average education quality, which make it an ideal location for conducting IVR experiments. Second, students in their seventh year of education are transitioning from primary to middle school. This period is characterized by the emergence of social challenges that require them to adapt to a new environment and a change in roles. Since students were from the same grade and had similar education and family backgrounds, they were of similar ages and had comparable technical experiences and cultural beliefs. Therefore, we did not consider these factors as moderating variables. With this context in mind, the objective of this study is to investigate the mechanism influencing students’ social experience in an IVR collaborative environment.

We screened the survey data to remove invalid responses, and a total of 87 valid questionnaire responses were ultimately retrieved. The following exclusion criteria were applied: (1) questionnaires with missing answers or completed with identical answers for each item; (2) students who did not participate in the SEC pre-test; and (3) students who did not participate in the post-test of technology acceptance, presence, collective efficacy, group effectiveness, and social experience. Among the 87 participants, 43 were male and 44 were female, with an age range of 12–13 years. All participants were given an informed consent form before the experiment, and parental consent was obtained. This study was also approved by the Institutional Review Board of Central China Normal University (CCNU-IRB-202306002a, approval date: 13 June 2023).

### 4.2. Determination of Sample Size

Two methods were employed to calculate the minimum sample size to ensure that the sample was sufficient for mediation analysis. Green [71] proposed N ≥ 50 + 8 m (where m refers to the number of predictors in the model) for determining the sample size for the coefficient of determination. Our hypothesized model consists of four independent variables, so the minimum sample size required is 82. A power analysis was also conducted using G*Power 3.1.9.4 according to the steps given by Memon et al. [72]. We used the F test to estimate the required sample size for the multiple linear regression used in this study. The following calculation parameters were as follows: the effect size was set as 0.15; the α error at 0.05; the power at 0.8; and the number of predictors at 4. The final minimum sample size was 85. The present study consists of 87 participants, which meets both the requirements above.

### 4.3. Research Procedure

**Figure 3:**
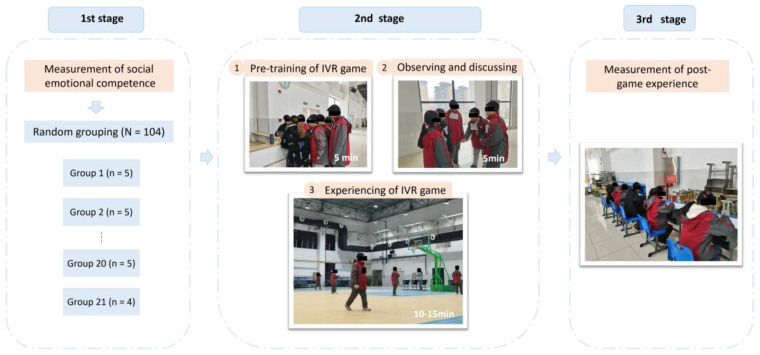
Research procedure.

The research procedure of this study consisted of three stages, as shown in Figure 3. In the first stage, participants were required to complete a questionnaire designed to assess their SEC on the day prior to the commencement of the experiment. The initial sample of 104 students was then randomly allocated into 21 groups of 4–5 students to participate in the collaborative IVR game. In the second stage of the experiment, students engaged in the collaborative IVR game within the virtual reality environment. The IVR experiment was conducted in a large indoor gymnasium to ensure that the students had sufficient space to move around. Before the experiment, the researchers allocated approximately 5 min to explain the rules and procedures to the participants. Students were then permitted to observe, discuss, and select their own roles in the IVR game. However, it was imperative that each group contained at least one defender, one attacker, and one collector. The duration of the IVR game was approximately 10–15 min. In the third stage, participants were given a post-game experience questionnaire after completing the IVR game.

### 4.4. Measures

Two research instruments were used: the questionnaire measuring participants’ SEC before the experiment and the post-game experience questionnaire, which measured participants’ technology acceptance, presence, IVR collaboration experience, and social experience.

#### 4.4.1. Instrument 1: SEC Questionnaire

The SEC scale was informed by the works of CASEL [73] and Zhou and Ee [74], with 25 items measuring 5 subscales of self-awareness, self-management, social awareness, relationship skills, and responsible decision-making. Each subscale contained 5 items (see Appendix A). All items were measured using a 5-point Likert scale, ranging from 1 (strongly disagree) to 5 (strongly agree).

#### 4.4.2. Instrument 2: Post-Game Experience Questionnaire

The post-game experience questionnaire consisted of four parts to measure participants’ technology acceptance, presence, IVR collaboration experience, and social experience (see Appendix B). According to Barrett, Pack and Quaid [22], the technology acceptance scale contained three subscales: PU, PEU, and ITU, and each subscale contained 3 items. The presence scale was derived from von der Pütten et al. [75] and comprised 8 items assessing students’ personal experiences in the virtual environment. The measurement of the IVR collaboration experience was based on the questionnaire developed by Vidergor [76]; the items from the original questionnaire were revised, resulting in the inclusion of two dimensions: collective efficacy (6 items) and group effectiveness (4 items). The scale of social experience was adopted from Vidergor [76] and contained 8 items.

### 4.5. Data Analysis

#### 4.5.1. Preliminary Analysis

Preliminary analysis was conducted to assess the descriptiveness, reliability, validity, and normality of the study variables. Descriptive analysis was assessed through mean and standard deviation (SD). Reliability was determined by Cronbach’s α. Normality was evaluated by the value of skewness and kurtosis. Moreover, confirmatory factor analysis (CFA) and correlational analysis were performed to examine the validity of the measured constructs, including convergent and discriminant validity. As suggested by Hair et al. [77], convergent validity was determined by factor loading, average variance extracted (AVE), and composite reliability (CR), while discriminant validity was assessed by comparing the square roots of AVE (√AVE) with correlation coefficients. Descriptive, reliability, normality, and correlation analyses were conducted using IBM® SPSS® software (version 21), and the CFA for the measured constructs was performed in IBM® SPSS® AMOS software (version 21).

#### 4.5.2. Mediation and Moderation Analysis

The mediation and moderation analysis was performed using PROCESS v4.2 macro (Model 90) to test the hypothesized serial mediation model [78]. PROCESS provides ordinary least squares regression-based path analysis using averages of indicators measuring each construct [78]. PROCESS does not require a high degree of correlation between variables, thereby reducing the likelihood of Type I errors. It is also particularly appropriate for empirical investigations involving a limited number of participants [79]. Several research studies have employed the PROCESS macro due to its robust ability to estimate indirect and conditional effects [80,81].

As shown in the moderated mediation model (Figure 1), the direct effect (c’) pertains to the association between X and Y, which accounts for the influence of all mediators. Conversely, a specific indirect effect signifies the relationship between X and Y mediated through a specific mediator or mediators. Values for technology acceptance were set as the predictor variable (X) and social experience as the outcome variable (Y). Multiple mediators were entered in the following order: presence, collective efficacy, and group effectiveness. Based on 5000 bootstrap samples, 95% bias-corrected confidence intervals (CI) were computed. The exclusion of zero within the 95% CI denoted a statistically significant mediating effect. We also incorporated gender and grade as covariates into the data analysis, thus preventing them from influencing the results.

#### 4.5.3. Model Fit Analysis

We conducted CFA to evaluate the model fit of the moderated mediation model in our study. The model fit indices examined included χ², df, χ²/df, RMSEA, CFI, TLI, and SRMR. Moreover, since the questionnaires were self-reports from a single data source, there is a possibility of introducing common method bias (CMB). Therefore, Harman’s single-factor test and CFA were used to determine CMB as suggested by Podsakoff [82]. Harman’s single-factor test commonly employs exploratory factor analysis (EFA) to assess CMB, in which all variables are loaded onto a single factor for the factor analysis. According to Podsakoff and Organ [83], if the variance explained by a single factor extracted using unrotated EFA does not exceed 40%, CMB should not be a significant concern. Additionally, CFA can also be utilized to assess CMB by constructing a single-factor model that includes all measurement items and calculating model fit indices. If the single-factor model demonstrates a poor fit and shows a significant difference in fit compared to a multifactor model, it is generally assumed that the presence of CMB is unlikely in the proposed model.

## 5. Results

### 5.1. Preliminary Analysis Results

The results of the reliability, validity, and normality of the measured constructs are listed in Table 1. As shown in Table 1, Cronbach’s α of the measured constructs was above 0.7, which indicated acceptable reliability for the measurement [84]. The results of normality test indicate that the skewness and kurtosis values of the data basically meet the requirements, with the absolute value of kurtosis less than 10, and the absolute value of skewness less than 3, indicating that the data exhibit approximately normal distribution characteristics. However, only ITU fails to meet the normal distribution, indicating that students generally have a willingness to continue using VR in the future.

Construct validity was determined by convergent and discriminant validity. According to Campbell and Fiske [85], acceptable convergent validity requires a factor loading exceeding 0.7, composite reliability (CR) values surpassing 0.6, and AVE values greater than 0.5. As seen in Table 1, most of the values met the requirements, thus indicating the acceptable convergent validity of the constructs. In addition, good discriminant validity requires that √AVE be greater than the correlation coefficients with other constructs [86]. As seen in Table 2, these values ranged from 0.574 to 0.983, which is greater than the correlation coefficients with other constructs, and thus met the requirements. Overall, the measured constructs were all found to be reliable and valid.

### 5.2. Mediation Analysis Results

**Figure 4:**
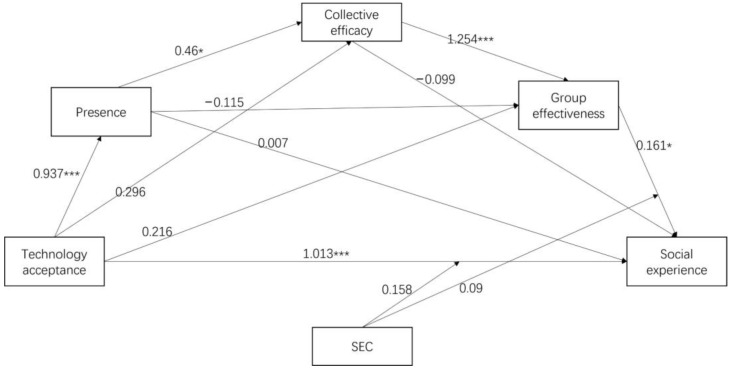
Research model predicting social experience: Path coefficients. Note. Non-standardized coefficients are displayed above. * *p* < 0.05, *** *p* < 0.001.

We tested whether presence, collective efficacy, and group effectiveness mediated the relationship between technology acceptance and social experience after controlling for age and gender. Table 3 presents the results of the mediation analysis. The results confirm significant positive relationships between technology and presence; presence and collective efficacy; collective efficacy and group effectiveness; technology acceptance and social experience, group effectiveness, and social experience. Table 4 demonstrates the indirect effects of technology acceptance on social experience. The results showed a significant indirect effect serially via presence, collective efficacy, and group effectiveness. Thus, H1 and H8 were supported, but H2, H3, H4, H5, H6, and H7 were not supported. The path of technology acceptance → presence → collective efficacy → group effectiveness → social experience generated a positive compound effect (as seen in Figure 4).

### 5.3. Moderation Analysis Results

**Figure 5:**
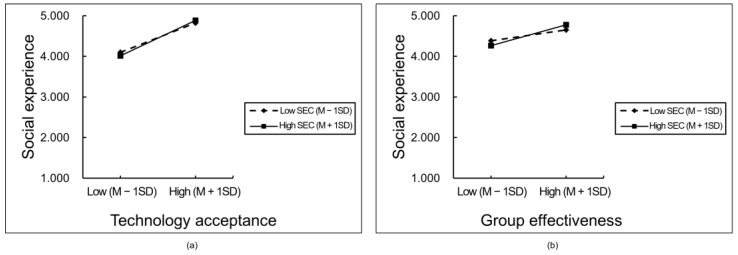
Moderation effect of social emotional competence on technology acceptance, group effectiveness, and social experience. Note: M − 1SD: the mean (M) minus one standard deviation (SD), indicating a level one standard deviation below the mean; M + 1SD: the mean (M) plus one standard deviation (SD), indicating a level one standard deviation above the mean. (**a**) the moderating effect of SEC on the relationship between technology acceptance and social experience; (**b**) the moderating effect of SEC on the relationship between group effectiveness and social experience.

We tested the moderating effect of SEC on (1) the association between technology acceptance and social experience and (2) the association between group effectiveness and social experience. However, the results showed that SEC does not moderate these two pathways. Figure 5 illustrates the moderating effect of SEC. As illustrated in Figure 5a, students with high and low SEC levels exhibited different levels of technology acceptance. It can be posited that the higher the level of technology acceptance, the more positive the students’ social experience. Students with high SEC and high technology acceptance had the best social experience, while students with high SEC and low technology acceptance had the worst social experience. As shown in Figure 5b, students with high and low SEC levels also exhibited different levels of effectiveness, with the higher group effectiveness demonstrating enhanced social experience. Students with high SEC and high group effectiveness had the best social experience, while students with high SEC and low group effectiveness had the worst social experience.

### 5.4. Model Fit Indices

The CFA showed an acceptable fit of the six moderated mediation models in our study. Moreover, the results of CFA showed that the one-factor model fit (χ^2^ = 4585.102, df = 1710, χ^2^/df = 2.681, RMSEA = 0.140, CFI = 0.280, TLI = 0.254 and SRMR = 0.153) was poorer than the fit of six-factor model (χ^2^ = 1178.452, df = 553, χ^2^/df = 2.131, RMSEA = 0.075, CFI = 0.731, TLI = 0.71, and SRMR = 0.097). The results of Harman’s single-factor test showed that 15 factors with eigenvalues greater than one were obtained without rotation. The first factor explained 23.657% of the total variance (lower than 40%), which indicates that there is no single factor that can explain most of the variance in the data, suggesting that the common method bias is minimal. Therefore, we concluded that common method variance did not have a significant impact on the study results.

## 6. Discussion

The findings of this study underscore the advantages of immersive virtual reality (IVR) in enhancing students’ social experiences, which is consistent with previous research studies [61,87]. By providing immersive and interactive virtual environments, IVR can facilitate the development of social identity and promotion of social interaction within [87]. Also, IVR can create unique opportunities for students to engage in collaborative tasks with improved communication opportunities [88]. A key contribution of this study, compared to prior research, is the identification of a key pathway demonstrating how collaboration in IVR can lead to enhanced social experience, offering valuable insights into the design of future IVR-based educational environments. Furthermore, our study reveals the equitable usability of the IVR environment, indicating that students across different levels of SEC can benefit from the IVR experience.

### 6.1. A Key Factor and Pathway Influencing Social Experience in IVR Collaboration

Consistent with previous research studies, technology acceptance shows a positive relationship with social experience [22,23]. PU and PEU influenced users’ ITU and are thus motivational aspects in technology-enhanced environments. Students who find the IVR environment easy to use tend to invest time and effort in collaboration in the IVR game. This positive attitude can foster participation and engagement in the virtual environment, enhancing students’ social experience. This finding aligns with a previous study on industrial operations, showing that students with high technology acceptance are more likely to demonstrate strong engagement during the training process [89].

This study revealed that the presence of IVR can influence social experience through collective efficacy and group effectiveness. The most salient pathway proved to be technology acceptance → presence → collective efficacy → group effectiveness → social experience. The immersive collaborative environment provided students with a sense of identity, social interaction, and co-presence which improved their collective efficacy and group effectiveness. Previous research studies have similar findings showing that the technological features of IVR can foster improved collaboration among students [90,91]. Regarding the relationship between IVR collaboration experience and social experience, many researchers had comparable observations that groups with high efficacy were typically capable of fostering constructive behavior, such as effective collaboration, communication, and conflict resolution [92,93]. These behaviors enable students to complete tasks efficiently and thus enhance their social experience. 

### 6.2. Social–Emotional Competence as a Moderator

Surprisingly, our research model found that SEC showed no significant moderating effect on the relationship between technology acceptance and social experience. Also, the results revealed no moderating effect of SEC in the relationship between group effectiveness and social experience. Common challenges that low-SEC students face in the real world often do not carry over into the virtual environment. According to social determination theory, individuals obtain three basic psychological needs in a social environment: competence, autonomy, and relatedness [94]. In collaborative IVR games, students can satisfy their needs for autonomy by freely choosing roles and modes of interaction, fulfill their needs for competence by completing tasks and obtaining rewards, and meet their needs for belongingness by collaborating with teammates. The characteristics of the IVR environment enabled low-SEC students to benefit from it and reduced the influence of the SEC. The high level of immersion and imagination provided by IVR technology enabled all participants to obtain a relatively enriched social experience. Moreover, the co-presence and flexibility of role identities within the IVR environment fostered social identification and a sense of belonging within the group, further mitigating the differences in SEC among individuals [95].

### 6.3. Biological and Psychological Perspectives on the Findings

The findings of this study can be interpreted through biological and psychological characteristics of seventh-grade students. From a biological perspective, students in early adolescence, characterized by heightened sensory processing sensitivity, tend to demonstrate enhanced responsiveness to various stimuli (e.g., visual and auditory cues) and respond positively to interventions [96,97]. Such heightened sensory stimulation is more likely to elicit a physiological sense of immersion, realism, and presence, which significantly enhances their social experience [98,99]. Furthermore, the IVR collaborative game in this study requires substantial physical activities such as running, shooting, and blocking. Previous research studies have shown that moderate physical activity can effectively contribute to student’s cognitive and emotional arousal [100,101] through enhanced neurogenesis and angiogenesis processes [102] and release of endorphins [103], which in turn can lead to enhanced social performance and experience. 

From a psychological perspective, seventh-grade students are at an important stage for developing cohesive personal identity, including self-awareness and self-identity [104]. Also, they exhibit a heightened need for social identification and interpersonal relationships, making collaboration and interaction within a VR environment particularly appealing [105,106]. Engaging with peers in an IVR setting fulfills adolescents’ need for social interaction by providing a safe space to explore interpersonal relationships, thereby enhancing the effects of collective efficacy and group effectiveness. Additionally, students at this age often demonstrate a stronger acceptance of technology innovations, such as virtual avatars and computer-mediated communications, which can help us understand students’ performance and experiences in technology-supported collaborative learning environments [107].

### 6.4. Caveats for Using IVR

Despite the potential benefits of using IVR for multi-user collaboration, some caveats need to be noted. Firstly, this study does not imply that IVR can replace traditional settings in enhancing students’ social experience. Rather, IVR can serve as a potential supplementary learning space, where schools can undertake IVR-related activities when financial and other conditions permit. Secondly, the utilization of IVR may also induce certain issues such as dizziness, technology fatigue, and social isolation. Hence, researchers should seek to achieve a balance between virtual and real-life experiences in their designs, minimizing system redundancy and complexity. Finally, it should be noted that some students may require prolonged adjustment when working in an IVR environment, which may entail tasks such as orientation, attention division, and progress tracking. Therefore, it is crucial to provide students with training prior to IVR programs and offer guidance throughout the experience with post-experience debriefing and reflection in a real-world context.

### 6.5. Practical Implications

Based on our results, several practical implications to promote social experience in IVR can be proposed. First, we consider that IVR might be a useful tool for social–emotional learning among adolescents because IVR has been demonstrated to be an effective medium to generating social experience. However, some caveats should be noted, such as motion sickness, technology fatigue, and cost effectiveness. Second, efforts should be made to promote students’ technology acceptance of the IVR environment to deliver social experience. Developers need to make IVR systems natural and intuitive to promote PEU. Teachers should arrange pre-training and supporting instruction before the intervention starts to inform students of the usefulness of IVR. Third, we recommend enhancing students’ presence through technology and instructional design, as presence is an essential element in the key path, for example, by enriching the vividness and interaction of IVR, we can improve students’ sense of presence. By improving the mapping of virtual and physical worlds, we can also further enhance presence through increased co-presence. Finally, as SEC does not moderate social experience in IVR, teachers can engage students in IVR broadly without concerns about previous SEC gaps. This ensures that all students can benefit equally from the social experience provided by IVR.

### 6.6. Limitations and Future Research Perspectives

There are several limitations of this study that should be addressed. First, data for this study were collected through self-reported questionnaires and may, therefore, suffer from a degree of subjectivity and bias. It is recommended that future research incorporate more objective data forms, such as observational and physiological data, to produce more accurate findings. Second, it should be noted that the study sample was quite small and was from one grade level in a specific school with limited representativeness. Factors like age, technology experience, and culture may have an impact on students’ IVR social experience. Future research should therefore be conducted with a more diverse sample and in a broader range of contexts to increase the generalizability and credibility of the findings. Third, to minimize disruption to the normal teaching process in the school, this study was conducted over a relatively short period of time, overlooking the long-term engagement and sustainability of IVR. This approach may have compromised the credibility of the findings. We encourage future researchers to conduct more longitudinal studies to verify our findings. Fourth, students choose the roles in IVR game freely based on their own interests and skills may also introduce various biases, including role stereotype bias, self-perception bias, social bias, and role selection conflicts, which in turn influence their social experience. Future research should focus on ensuring role design equality, providing students with multiple options within the IVR environment to facilitate the making of rational choices.

This study provides several insights for future research. First, future research should include more replication studies to validate the key pathway identified in this study across diverse educational contexts with longitudinal evidence. Second, subsequent studies could consider incorporating diverse physiological data (e.g., galvanic skin response, heart rate, etc.) and psychological measurements (mental health, social–emotional competence) to expand and optimize the established mechanism of social experience generation in IVR environments. Finally, based on the finding that IVR can enhance social experience, future research could further investigate how the enhanced social experience in IVR can be translated into improved mental health and social emotional development for children and adolescents through effective design of the IVR space and collaborative mechanism.

## 7. Conclusions

Employing the SLAM-based inside-out tracking technique, we designed a multi-user IVR collaborative game and conducted an exploratory study that offers alternative possibilities for enhancing social experiences for seventh graders in a virtual environment. The study aims to investigate the mechanisms underlying the relationship between technology acceptance and social experience, considering the influence of presence, collective efficacy, and group effectiveness. Technology was found to be significantly related to social experience. The results of the mediation analysis indicated the existence of a significant pathway between the variables of presence, collective efficacy, group effectiveness, and social experience. No significant moderating effects of SEC were found for the relationship between technology acceptance and social experience, group effectiveness, and social experience.

## Figures and Tables

**Table 1:** Reliability, validity, and normality indicators of the measured constructs.

Constructs	Items	Factor Loading	Cronbach’s α	CR	AVE	Skewness	Kurtosis
Technology acceptance	9		0.883			−2.869	13.005
Perceived usefulness	3	[0.836–0.891]	0.903	0.906	0.762	−1.788	3.354
Perceived ease of use	3	[0.786–0.852]	0.846	0.851	0.655	−2.107	6.789
User’s behavioral intention to use	3	[0.711–0.916]	0.849	0.860		−4.659	27.499
Presence	8	[0.401–0.814]	0.774	0.788	0.329	−2.199	7.317
Collective efficacy	6	[0.447–0.942]	0.852	0.852	0.504	−1.771	3.329
Group effectiveness	4	[0.898–0.962]	0.965	0.875	0.966	−1.555	1.182
Social experience	8	[0.530–0.833]	0.878	0.523	0.897	−1.660	2.469
Social–emotional competence	25		0.896			−0.620	1.555
Self-awareness	5	[0.417–0.715]	0.736	0.758	0.391	−1.224	3.089
Self-management	5	[0.563–0.794]	0.842	0.847	0.530	−0.495	0.103
Social awareness	5	[0.558–0.748]	0.801	0.804	0.453	−0.223	−0.601
Relationship skills	5	[0.343–0.679]	0.678	0.709	0.338	−1.176	1.813
Responsible decision-making	5	[0.638–0.753]	0.818	0.820	0.478	−1.091	1.279

Note: CR, composite reliability; AVE, average variance extracted.

**Table 2:** Correlations, descriptive statistics, and discriminant validity of the measured constructs.

Constructs	1	2	3	4	5	6	7	8	9	10	11	12
1 PU	**0.873**											
2 PEU	0.463 **	**0.809**										
3 ITU	0.494 **	0.489 **	**0.820**									
4 Presence	0.613 **	0.489 **	0.588 **	**0.574**								
5 CE	0.291 **	0.349 **	0.349 **	0.488 **	**0.710**							
6 GE	0.310 **	0.346 **	0.279 **	0.395 **	0.813 **	**0.983**						
7 SE	0.778 **	0.459 **	0.604 **	0.563 **	0.425 **	0.487 **	**0.937**					
8 SEA	0.203	0.204	0.188	0.305 **	0.147	0.089	0.185	**0.625**				
9 SM	0.169	0.176	0.270 *	0.328 **	0.422 **	0.309 **	0.125	0.482 **	**0.728**			
10 SOA	0.178	0.199	0.163	0.309 **	0.218 *	0.061	0.232 *	0.530 **	0.378 **	**0.673**		
11 RS	−0.022	−0.012	−0.165	−0.133	0.000	−0.016	0.014	0.464 **	0.358 **	0.389 **	**0.581**	
12 RD	0.009	−0.066	−0.058	0.043	0.057	−0.007	0.037	0.454 **	0.339 **	0.580 **	0.475 **	**0.691**
**Mean**	4.609	4.588	4.816	4.278	4.308	4.170	4.533	4.205	3.581	3.468	3.956	3.679
**SD**	0.613	0.577	0.503	0.601	0.793	1.215	0.621	0.682	0.791	0.881	0.773	0.742

Note: PU, perceived usefulness; PEU, perceived ease of use; ITU, user’s behavioral intention to use; CE, collective efficacy; GE, group effectiveness; SE, social experience; SEA, self-awareness; SM, self-management; SOA, social awareness; RS, relationship skills; RD, responsible decision-making; Diagonal elements in bold are the square root of AVE; * *p* < 0.05; ** *p* < 0.01.

**Table 3:** Results of mediation analysis.

							95%CI	
Model	R^2^	F	B	SE	T	*p*	Lower	Upper
Outcome: Presence	0.511	28.967						
Constant			6.46	1.404	4.6	0.000	3.666	9.253
Technology acceptance			0.937	0.103	9.127	0.000	0.733	1.141
Age			−0.194	0.115	−1.689	0.095	−0.423	0.034
Gender			0.125	0.092	1.357	0.178	−0.058	0.308
Outcome: Collective efficacy	0.272	7.673						
Constant			4.407	2.549	1.728	0.088	−0.665	9.478
Technology acceptance			0.296	0.235	1.257	0.212	−0.173	0.764
Presence			0.46	0.178	2.587	**0.011**	0.106	0.814
Age			−0.196	0.189	−1.035	0.304	−0.573	0.181
Gender			0.216	0.151	1.434	0.155	−0.084	0.516
Outcome: Group effectiveness	0.675	33.659						
Constant			−8.021	2.673	−3.001	0.004	−13.34	−2.702
Technology acceptance			0.216	0.245	0.884	0.379	−0.271	0.704
Presence			−0.115	0.191	−0.603	0.548	−0.494	0.264
Collective efficacy			1.254	0.114	11.027	**0.000**	1.028	1.481
Age			0.263	0.196	1.338	0.185	−0.128	0.653
Gender			−0.063	0.157	−0.401	0.689	−0.376	0.25
Outcome: Social experience	0.649	15.842						
Constant			5.347	1.551	3.447	0.001	2.259	8.436
Technology acceptance			1.013	0.147	6.876	0.000	0.72	1.307
Presence			0.007	0.108	0.063	0.95	−0.208	0.222
Collective efficacy			−0.099	0.102	−0.967	0.336	−0.301	0.104
Group effectiveness			0.161	0.062	2.606	**0.011**	0.038	0.283
SEC			0.003	0.082	0.032	0.974	−0.161	0.166
Technology acceptance × SEC			0.158	0.245	0.647	0.52	−0.329	0.646
Group effectiveness × SEC			0.09	0.083	1.088	0.28	−0.075	0.255
Age			−0.051	0.109	−0.472	0.638	−0.269	0.166
Gender			0.13	0.087	1.493	0.139	−0.043	0.304

Note: SEC, social–emotional competence; SE, standard error; CI, confidence interval; *p* values significant at 0.05 level are bolded.

**Table 4:** Indirect effects of technology acceptance on social experience via mediators.

			95% CI	
Path	Effect	BootSE	Lower	Upper
H5: X → M1 → Y	0.006	0.129	−0.254	0.263
H2: X → M2 → Y	−0.029	0.059	−0.155	0.092
H3: X → M3 → Y	0.046	0.099	−0.034	0.348
H6: X → M1 → M2 → Y	−0.042	0.067	−0.192	0.080
H7: X → M1 → M3 → Y	−0.023	0.053	−0.168	0.042
H4: X → M2 → M3 → Y	0.079	0.108	−0.078	0.258
H8: X → M1 → M2 → M3 → Y	0.115	0.074	0.001	0.293

Note: X: technology acceptance, M1: presence, M2: collective efficacy, M3: group effectiveness, Y: social experience. Confidence intervals (95% CI) that contain zero are interpreted as non-significant.

## Data Availability

Data can be retrieved from Mendeley Data at https://www.doi.org/10.17632/36j5j962ts.1 (accessed on 16 August 2024).

## Appendix A. Social–Emotional Competence (SEC) Questionnaire

Self-awareness

I know what I am thinking and doing.

I understand why I do and what I do.

I understand my moods and feelings.

I know when I am moody.

I can read people’s faces when they are angry.

Social Awareness

I can recognise how people feel by looking at their facial expressions.

It is easy for me to understand why people feel the way they do.

If someone is sad, angry or happy, I believe I know what they are thinking.

I understand why people react the way they do.

If a friend is upset, I have a pretty good idea why.

Self-management

I can stay calm in stressful situations.

I can stay calm and overcome anxiety in new or changing situations.

I can stay calm when things go wrong.

I can control the way I feel when something bad happens.

When I am upset with someone, I will wait till I have calmed down before discussing the issue.

Relationship Management

I will always apologise when I hurt my friend unintentionally.

I always try and comfort my friends when they are sad.

I try not to criticise my friend when we quarrel.

I am tolerant of my friend’s mistakes.

I stand up for myself without putting others down.

Responsible Decision-making

When making decisions, I take into account the consequences of my actions.

I ensure that there are more positive outcomes when making a choice.

I weigh the strengths of the situation before deciding on my action.

I consider the criteria chosen before making a recommendation.

I consider the strengths and weaknesses of the strategy before deciding to use it.

## Appendix B. Post-Game Experience Questionnaire

Technology acceptance

*Perceived usefulness (PU)*

VR games have helped me better understand how to collaborate.

VR games can improve my collaborative abilities.

Participating in VR games has enabled me to learn useful collaboration skills.

*Perceived ease of use (PEU)*

I found this VR game easy to use.

Operating this VR game is easy for me.

Learning how to operate this VR game is easy for me.

*User’s behavioral intention to use (ITU)*

I consider this VR game as an interesting environment for experiencing.

I think this VR game can enhance my willingness to use VR.

If given the opportunity, I would like to play this VR game again.

Presence

At the start of the game, I felt as if I were entering a new world.

While playing the game I felt as if I were part of a game that was not part of the reality.

All my senses were stimulated by the game experience.

I feel the existence of virtual objects in the game.

The virtual objects felt real to me.

During the game, I often forget that it is a computer-generated virtual scene.

I was aware of the presence of my teammates while playing the game.

I was able to easily communicate with my teammates while playing the game.

VR collaboration experience

*Collective-Efficacy*

My group could pull itself out of difficult situations.

I believe that failure will motivate our group to work harder.

My team members worked harder than expected.

My group completed its task successfully.

My group accomplished the task effectively.

I believe that the members of my group know how to solve problems.

*Group Effectiveness*

My group completed the task with excellence.

My group effectively fulfilled the task requirements.

My group accomplished the objectives with complete satisfaction.

My group smoothly completed the task.

*Social Experience*

I felt I was not alone.

I felt the group members supported me.

I felt I had someone to work with in my group.

I felt like a member of my group.

I felt connected to others in my group.

I think completing tasks with group members is a social experience.

I think I can share things with group members.

This game influenced me because it involves teamwork.
